# Prominence of work versus other stressors among construction industry workers

**DOI:** 10.1093/occmed/kqag040

**Published:** 2026-05-19

**Authors:** Andrew F Arena, Daniel Collins, Bojana Vilus, Samuel B Harvey, Mark Deady

**Affiliations:** Black Dog Institute, Faculty of Medicine and Health, University of New South Wales, Hospital Road, Randwick, NSW, 2031, Australia; Black Dog Institute, Faculty of Medicine and Health, University of New South Wales, Hospital Road, Randwick, NSW, 2031, Australia; Black Dog Institute, Faculty of Medicine and Health, University of New South Wales, Hospital Road, Randwick, NSW, 2031, Australia; Black Dog Institute, Faculty of Medicine and Health, University of New South Wales, Hospital Road, Randwick, NSW, 2031, Australia; Black Dog Institute, Faculty of Medicine and Health, University of New South Wales, Hospital Road, Randwick, NSW, 2031, Australia

## Abstract

**Background:**

Considerable research has been dedicated to identifying the presence and impact of workplace psychosocial risk factors in the construction industry; however, there is limited qualitative evidence that contextualizes workplace stressors in relation to broader life stress.

**Aims:**

To qualitatively explore the perceived prominence of work stressors in relation to other stressors in construction industry workers’ lives.

**Methods:**

Workers from an Australian construction company participated in a nation-wide survey. An open-ended question was asked regarding the greatest source of stress workers face within or outside of the workplace. An inductive, manifest content analysis was conducted using a qualitative descriptive approach to meaningfully categorize workers’ responses.

**Results:**

Responses from 608 workers were analysed. Stressors in the work-related category were most commonly reported as workers’ most significant source of stress (45%), followed by combined work and home/personal stressors (35%), home or personal stressors (14%) and no stressors (6%). Collectively, work was implicated in 80% of workers’ most prominent source of stress. The most prominent specific stressor was work overload (high job demands, pressure and long hours often reported together; 23%), followed by poor work-life balance (18%).

**Conclusions:**

Work-related stressors are not only highly prevalent among construction industry workers, but are much more commonly described as their most prominent source of stress compared to home or personal factors. The centrality of work-related stressors within workers’ lives more broadly, including those that straddle the work-home divide (particularly poor work-life balance), suggests the need for holistic approaches to optimally address mental health.

## Teaser

How workers in the construction industry view the relative prominence of stressors within and outside of the workplace was explored using a descriptive qualitative approach. Work was implicated in 80% of workers’ self-described greatest source of stress, indicating how central work-related stressors are in workers’ lives more broadly. Work overload and poor work-life balance were most commonly reported as the greatest specific stressors workers face, by 23% and 18% of workers, respectively.

## Introduction

Construction is both one of the largest industries in the world and one that is particularly predisposed to mental ill-health, suicide and psychosocial risks [1–5]. Competitive tenders, tight deadlines and a hypermasculine workplace culture contribute to psychologically harmful working conditions, including long hours, excessive job demands, bullying, poor supervisor and colleague support and high job insecurity [1,6–10]. Beyond workplace-specific risk factors, there is now considerable evidence demonstrating the negative impacts of poor work-life balance within construction [11].

Substantial evidence has both identified the presence of these psychosocial risk factors among construction industry workers [9,12,13] and linked them to increased risk of mental ill-health [14,13]. This research has relied heavily upon quantitative methodologies, and what is lacking is qualitative evidence that explores how these workers perceive the relative prominence of work stress within the broader context of their lives. Such research would improve understanding of the types of stressors that workers themselves identify as most important to them, helping to contextualize the literature on work-related risks and inform occupational mental health interventions. This study aimed to explore the perceived prominence of work stressors in relation to other stressors in construction industry workers’ lives.

## Methods

All employed and subcontracted workers of a large, national Australian construction company (approximately 4000) were invited to participate in an online survey in September 2022, via e-mail and flyers distributed on construction sites. Participation was voluntary, anonymous and required informed consent. The study was conducted in accordance with the Declaration of Helsinki, and all methods were approved by the University of New South Wales Human Research Ethics Committee (HC220372). The population sampled was comprised of approximately 50% tradespeople and 75% men. Workers had access to varied stress management resources, including an employee assistance program, daily mindfulness activities and mental health monitoring and referral tools.

Within a larger quantitative study on workplace psychosocial risk factors [13], workers were asked an open-ended question to contextualize the relative importance of workplace and other sources of stress in workers’ lives. This question regarded participants’ perceived greatest source of stress: “In your own words, what is the most significant stress you face at work or at home?”

An inductive, manifest content analysis [15] was undertaken using a qualitative descriptive approach [16], organizing responses into meaningful descriptive categories. A subset of 25% of responses was double-coded and discussed to refine the approach to coding before the lead author coded the remaining responses. Categorization was refined using triangulation between three authors. Given the nature of the question, each participant’s response was coded only once to capture the category of their greatest source of stress.

## Results

A total of 710 workers participated (608 with valid open-ended responses), representing an approximate response rate of 18% of invited workers. The qualitative sample was predominantly men (69%), university educated (60%), Caucasian/European (72%) and employed full-time (93%). The mean age was 37.9 years (SD = 11.0, range = 18–74). Occupational roles spanned technical/analytical workers (e.g. engineers, surveyors; 35%), office workers (30%), managers (21%) and tradespeople (14%).

The average response length was 7.2 words (SD = 8.0, range 1–55). Four superordinate categories of responses were identified: work-related stressors, home or personal stressors, combined work and home/personal stressors and no stressors (Table 1, see Supplementary Materials for data split by demographic subgroups).

**Table 1:** Content analysis of workers’ greatest source of stress alongside illustrative quotes

Content category	*n* (%)	95% CI	Example quotes
1. Work-related stressors	272 (45)	41–49%	
1.1. Work overload (high job demands, pressure, and long hours)	139 (23)	20–26%	“Long working hours, job demands” “Work overload”
1.2. Interpersonal issues and lack of support at work	55 (9)	7–12%	“Conflict with superior” “Working with difficult people”
1.3. Uncertainty around one’s own capabilities/new to job	11 (2)	1–3%	“Feeling that I am not good enough to do my job” “New role, uncertain of my place”
1.4. Job insecurity	8 (1)	1–3%	“Not knowing if I will have work”
1.5. Organizational systems or upper management	7 (1)	<1–2%	“Poor upper management decisions” “Systems that don’t support the role I am doing”
1.6. Long commute	5 (1)	<1–2%	“Travel to/from work”
1.7. General/unique/multiple distinct work stressors (including role ambiguity/conflict, specific work tasks/issues, generic ‘work’ stress)	47 (8)	6–10%	“Not having a business as usual” “Unions” “Staff turnover… Long hours, covering for others when on leave/sick, keeping team together.”
2. Home or personal stressors	87 (14)	12–17%	
2.1. Personal mental health and well-being issues (including sleep)	24 (4)	3–6%	“Existential crises” “My low self-esteem and overthinking”
2.2. Family-related stressors	23 (4)	2–6%	“Kids” “Wellbeing of family (parents)”
2.3. Partner-related stressors	18 (3)	2–5%	“Marriage break up” “Health of my partner”
2.4. General/unique home/personal issues (including study stressors, physical health issues, generic “home” stress)	22 (4)	2–5%	“Personal problems” “Delayed home project”
3. Combined work and home/personal stressors	210 (35)	31–38%	
3.1. Poor work-life balance (struggling to balance competing demands/priorities of work and home life, not having enough time for oneself outside of work)	108 (18)	15–21%	“Finding the time to do my job, be present at home for my wife, and also find time for myself” “Juggling home life with long working hours”
3.2 Not enough time in one’s life in general	35 (6)	4–8%	“Managing my time” “Not enough time in a day”
3.3 Financial stressors (being both work-related and impacting home life)	31 (5)	3–7%	“Providing financial security for my family” “Ability to support myself on a single wage”
3.4 Interpersonal issues across contexts	9 (2)	1–3%	“Relationships at home and work” “Trying to keep everyone happy”
3.5. General combined or multiple unique work and home stressors (including general life stress)	27 (4)	3–6%	“Sometimes just making it to the next day” “High work load, sick mother”
4. No stressors	39 (6)	5–9%	“Nothing” “I don’t get stressed”

*N *= 608 participants with valid responses used in these analyses (analyses excluded 49 participants with no response and 53 responses too ambiguous to be coded). Sub-categories with <5 responses coded within them were combined within the general categories in each domain.

Work-related stressors were the most common superordinate category (45%), and the most prominent specific stressor related to work overload (high job demands, pressure and long hours typically reported together), reported by almost a quarter of workers (23%). Specific home or personal stressors were much less commonly reported as most significant (14%). However, analyses revealed the prominence of stressors that straddle the work-home divide (35%). In particular, the second most prominent single stressor was poor work-life balance, reported by over one in six workers (18%). These workers struggled to maintain boundaries between work and home domains, to meet their “competing” priorities, and to “juggle” the large time commitments of work and home life while still finding time to relax and pursue personal or social goals.

## Discussion

The current findings extend existing research on the psychosocial risk factors present in the construction industry by elucidating their relative prominence in the broader context of workers’ lives. When including the combined work-home category, work was implicated in 80% of workers’ most significant source of stress. The specific stressors self-described by workers largely reflect the range of stressors identified in the literature [9,12,14]. Interestingly, the two factors workers most commonly reported as their greatest source of stress, work overload and poor work-life balance, reflect the two most frequently researched stressors in the literature [12]. Work overload is known to be a major contributor to work-life conflict [17], and work-life conflict negatively impacts workplace well-being and productivity [11], indicating that these prominent stressors likely compound each other. These findings, therefore, call upon occupational health practitioners and researchers to address construction workers’ stress in a holistic manner that extends beyond conditions within the workplace. Flexibility initiatives show promise in improving work-life balance, although these can be challenging to implement effectively given the nature of construction work (e.g. competitive project tenders with client-driven deadlines; 11,18,19], suggesting this is a priority for future investigation.

While strengths of this study include the sample size and nation-wide reach, an important limitation is that workers were sampled from a single organization that may not represent the entire industry. Furthermore, the low survey response rate (particularly among tradespeople) suggests current results may not reflect all workers, and assessing the sample’s representativeness is hindered by the restricted demographic data available for the total population. Future research could benefit from more in-depth qualitative investigation of the relative contributions of work and non-work stressors, how these may hinge upon diverse personal circumstances, and their relationships with important work outcomes (e.g. absenteeism).

This study reveals that work-related stressors in the construction industry are not only important within the workplace but are also typically the most central source of stress in workers’ lives, more prominent than home or personal factors unrelated to work.

Key learning points

**What is already known about this subject:** 

- Workers in the construction industry are at high risk of mental ill-health and suicide.
- There is considerable published evidence pertaining to the presence and negative impacts of workplace psychosocial risk factors within the construction industry.
- There is limited qualitative research in this area, particularly that which investigates the prominence of these work-related stressors relative to other stressors in workers’ lives.
- **What this study adds:** 
- When given the opportunity to freely describe their greatest source of stress, 80% of construction industry workers reported work-related or combined work and home factors.
- Work overload (excessive job demands, pressure and long hours) was reported by close to 1 in 4 workers as their most significant specific stressor, and poor work-life balance was reported by over 1 in 6 workers.
- Factors pertaining to work are therefore not only stressful, but are much more commonly described as the most prominent source of stress for workers compared to home or personal factors.
- **What impact this may have on practice or policy:** 
- From workers’ perspectives, work overload and poor work-life balance are likely to be the most important specific intervention targets to improve stress.
- Given the relative prominence of work-related stressors within construction industry workers’ lives, upstream work design initiatives that directly address workplace conditions (rather than solely individual-level interventions) are a priority for occupational health practitioners.
- To benefit workers holistically, organizations, practitioners and policymakers should further explore how to overcome industry barriers to optimally implementing work-life balance initiatives.

## Supplementary Material

kqag040_Supplementary_Data

## Data Availability

The data that support the findings of this study can be made available from the corresponding author upon reasonable request.
