# Supplementary material for: Prominence of work versus other stressors among construction industry workers
Source: Occup Med (Lond). 2026 May 19;76(4):290–3. doi: 10.1093/occmed/kqag040 (PMC13412387; doi:10.1093/occmed/kqag040)
Supplement: kqag040_Supplementary_Data [file kqag040_supplementary_data.docx]

Prominence of work versus other stressors among construction industry workers

**Supplementary Materials**

**Table S1.**

| Demographic | | Work Factors | Home or Personal Factors | Combined Work and Home Factors | No Stressors |
| --- | --- | --- | --- | --- | --- |
|  |  | *n* (%) | *n* (%) | *n* (%) | *n* (%) |
| Age tertiles | 18–30 | 90 (33) | 30 (35) | 58 (28) | 7 (18) |
|  | 31–41 | 92 (34) | 35 (41) | 67 (32) | 19 (49) |
|  | 41–75 | 90 (33) | 21 (24) | 85 (40) | 13(33) |
| Gender | Man | 196 (72) | 52 (61) | 139 (67) | 31 (79) |
|  | Woman | 75 (28) | 33 (39) | 67 (33) | 8 (21) |
| Occupation | Tradespeople | 24 (10) | 19 (22) | 27 (13) | 12 (32) |
|  | Technical/Analytical | 106 (40) | 24 (28) | 73 (35) | 9 (24) |
|  | Office Workers | 71 (27) | 33 (39) | 61 (29) | 12 (32) |
|  | Managers | 66 (25) | 9 (11) | 47 (23) | 5 (13) |
| Employment | Full-time | 259 (96) | 75 (86) | 194 (93) | 36 (97) |
|  | Part-time/Casual | 12 (4) | 12 (14) | 15 (7) | ^a^ |
| Education | University | 182 (67) | 45 (52) | 124 (60) | 12 (31) |
|  | Less than university | 90 (33) | 41 (48) | 84 (40) | 27 (69) |
| Ethnicity | Caucasian/European | 188 (68) | 63 (71) | 160 (74) | 25 (71) |
|  | Asian | 50 (18) | 13 (15) | 35 (16) | 11 (31) |
|  | Middle Eastern | 21 (8) | ^a^ | 8 (4) | ^a^ |
|  | Other | 17 (6) | 10 (11) | 12 (6) | ^a^ |

*Workers’ Greatest Source of Stress Split by Demographic Subgroups*

*Note:* Data are not reported when there are <5 participants in a cell.
